# Effects of Heat Treatment Cooling Methods on Precipitated Phase and Tensile Properties of Fe-18Mn-10Al-1C-5Ni Lightweight Steel

**DOI:** 10.3390/ma18102364

**Published:** 2025-05-19

**Authors:** Yu Wang, Heng Cao, Yanchun Lou, Lei Cao, Yunbao Gao, Ling Zhao

**Affiliations:** 1 State Key Laboratory of Advanced Casting Technologies, Shenyang 110022, China; caohengheng123@gmail.com (H.C.); louych@chinasrif.com (Y.L.); gyb1394005869@163.com (Y.G.); zhaoling1214@126.com (L.Z.); 2 China Academy of Machinery Shenyang Research Institute of Foundry Co., Ltd., Shenyang 110022, China; 3 School of Materials and Metallurgy, University of Science and Technology Liaoning, Anshan 114051, China; cao_lei125@126.com

**Keywords:** Fe-Mn-Al-C-Ni lightweight steel, precipitating second phase, mechanical properties, fracture mechanism

## Abstract

This research focuses on Fe-18Mn-10Al-1C-5Ni lightweight steel and deeply explores the influences of three different cooling methods, namely, water quenching (WQ), air cooling (AQ), and furnace cooling (FQ), on the precipitation behavior of the B2 phases and κ-carbides in the lightweight steel. The intrinsic relationship among the precipitated phases, mechanical properties, and fracture behavior is revealed. Compared with the WQ sample, the size of the intragranular B2 phase in the AQ sample did not change significantly (an increment of 9 nm), but nano-sized κ-carbides appeared at the grain boundaries and inside the grains. The yield strength and tensile strength of the AQ sample significantly increased to 1232 MPa and 1347 MPa, respectively, while an elongation of 17.4% was still maintained, which benefitted from the synergistic effect of the grain boundary B2, intragranular B2, and nano-sized κ-carbides. When the cooling rate of the heat treatment was further reduced, the size of the intragranular B2 phase in the FQ sample increased slightly (332 nm), and the κ-carbides at the grain boundaries became obviously coarsened (170 nm), resulting in a severe reduction in the elongation (2.3%) because, during the tensile deformation process, the coarsened κ-carbides at the grain boundaries promoted the nucleation of voids and microcracks. The present work provides new insights into the cooling heat treatment process of lightweight steel.

## 1. Introduction

Fe-Mn-Al-C lightweight steels have garnered extensive attention owing to their unique combination of low density, excellent mechanical properties, and corrosion resistance [1,2,3,4]. The incorporation of 1 wt% aluminum into Fe-Mn-Al-C lightweight steels has been shown to reduce the steel density by approximately 1.3% [5]. Depending on the alloy composition, their microstructures typically comprise austenite, ferrite, and κ-carbides [1,6,7]. Although austenitic Fe-Mn-Al-C steel exhibits impressive ductility (20-80%), its relatively low yield strength (300–900 MPa) poses a significant limitation [8,9,10]. Consequently, strategies aimed at enhancing the yield strength without compromising the ductility are essential for the broader application of these materials, particularly in the automotive sector.

Among the various strengthening mechanisms, κ-carbide precipitation plays a critical role in improving the yield strength of Fe-Mn-Al-C lightweight steels [11,12]. Through the careful control of the heat treatment parameters, the size, distribution, and volume fractions of κ-carbides can be finely tuned, thereby tailoring the mechanical response [13,14]. Short-duration aging promotes the formation of nano-sized κ-carbides within austenite grains, substantially enhancing the yield strength while maintaining satisfactory ductility [12]. As the aging time increases, intragranular κ-carbides grow in both their size and volume fractions, leading to further improvements in the strength [14,15]. However, excessive aging can trigger eutectoid decomposition, resulting in the formation of coarsened κ-carbides at the austenite grain boundaries, severely degrading the ductility [14,16]. Thus, the precise control of the heat treatment process and the optimization of the κ-carbide precipitation behavior are crucial for maximizing the tensile properties of these steels. In addition to κ-carbides, the introduction of nickel into Fe-Mn-Al-C steels facilitates the formation of a NiAl-type B2 intermetallic phase [17]. This non-shearable phase markedly enhances the tensile strength and work hardening capability [18,19,20]. More importantly, the co-precipitation of κ-carbides in B2 phase-strengthened steel can lead to a synergistic dual-precipitation strengthening effect [20,21,22,23]. For instance, aging Fe-16.36Mn-8.94Al-0.84C-3.02Ni at 600 °C for just 5 min results in a significant yield strength increment (~300 MPa) while retaining excellent ductility (37.9%) [22]. Nonetheless, B2 phases can act as preferential nucleation sites for κ-carbides at the grain boundaries, promoting embrittlement upon prolonged aging; for example, the elongation decreased by 20.6% after 60 min [22]. Consequently, the meticulous design of the heat treatment protocols and stringent control over the size and spatial distribution of the κ-carbides in B2-strengthened steels are imperative for further optimizing their tensile performance. Despite these advancements, research exploring cooling methods to achieve an enhanced strength–ductility balance in Fe-Mn-Al-C-Ni steels remains relatively sparse. Moreover, comprehensive studies elucidating the precipitation behaviors—morphology, size distribution, location—of B2 phases and κ-carbides, and their collective impact on mechanical properties, are still lacking. Addressing this gap, the present study focused on Fe-18Mn-10Al-1C-5Ni lightweight steel. The primary objective was to realize an optimized microstructure featuring homogeneously distributed B2 phases at the grain boundaries alongside finely dispersed intragranular κ-carbides. The mechanisms underlying multiphase precipitation and their corresponding influence on the mechanical behavior are comprehensively discussed. The organization of the paper is as follows: Section 2 describes the preparation of the Fe-18Mn-10Al-1C-5Ni steel, the three cooling routes, and the tensile testing procedures. Section 3 presents the experimental results, covering the phase distribution, precipitate morphology, and engineering stress–strain behavior. Section 4 discusses how the cooling rate influences the B2 and κ-carbide precipitation, microstructure–property relationships, and fracture mechanisms. Finally, Section 5 summarizes the main findings and outlines potential implications for the lightweighting of automotive steels.

## 2. Materials and Methods

### 2.1. Materials and Processing

A 50 kg ingot of Fe-18Mn-10Al-1C-5Ni lightweight steel (actual chemical composition: Fe-18.20Mn-10.23Al-1.05C-5.04Ni, wt.%, as determined by spark optical emission spectroscopy) was synthesized using a vacuum induction melting furnace. The charge was heated from room temperature to 1600 °C at a rate of 10 °C/min and was held at 1600 °C for 30 min to ensure the complete dissolution and chemical homogeneity of all the alloying elements. The ingot was hot-forged within a temperature range of 1145–1200 °C into a billet measuring 500 × 120 × 70 mm. Subsequent hot rolling at 1200 °C produced sheets with a thickness of 5 mm, which were immediately subjected to water quenching (“WQ”). Solution treatment was performed at 1000 °C for 1 h, followed by another WQ step. The sheets were then cold-rolled to a final thickness of 1 mm, with a 2% thickness reduction per pass, and annealed at 950 °C for 10 min. Post-annealing cooling was conducted via three different routes: WQ, air quenching (AQ), and furnace quenching (FQ). The temperature range for the furnace quenching, water quenching, and air quenching was from 950 °C to room temperature (25 °C), with cooling times of 12 h, 2 s, and 5 min, respectively. Thus, the cooling rates of the furnace quenching, water quenching, and air quenching were 0.02 °C/s, 463 °C/s, and 3 °C/s, respectively.

### 2.2. Microstructural Characterization and Mechanical Testing

Microstructural characterization was carried out using scanning electron microscopy (SEM) (Zeiss Crossbeam550, Oberkochen, Germany), electron backscatter diffraction (EBSD) (Oxford symmetry, Oxford, UK), transmission electron microscopy (TEM) (JEOL 2100F, Tokyo, Japan), and scanning transmission electron microscope (STEM) (JEM-ARM200F, Tokyo, Japan) in high-angle annular dark field (HAADF) mode. EBSD measurements were performed at an accelerating voltage of 20 kV with a step size of 0.25 μm. TEM foils were prepared using a Tenupol-5 twin-jet electro-polisher (Struers (Shanghai) Ltd., Shanghai, China) in a solution of 90% acetic acid + 10% perchloric acid at 32 V. Tensile tests were conducted using a universal testing machine (JEOL, AG-XPLUS250KN, Tokyo, Japan) on dog-bone specimens with a gauge size of 25 × 10 × 1 mm aligned along the rolling direction. All tensile tests were performed at room temperature with a strain rate of 2 × 10^−3^ s^−1^, and each condition was tested in triplicate to ensure data reliability.

## 3. Results

### 3.1. Microstructure Characterization

**Figure 1:**
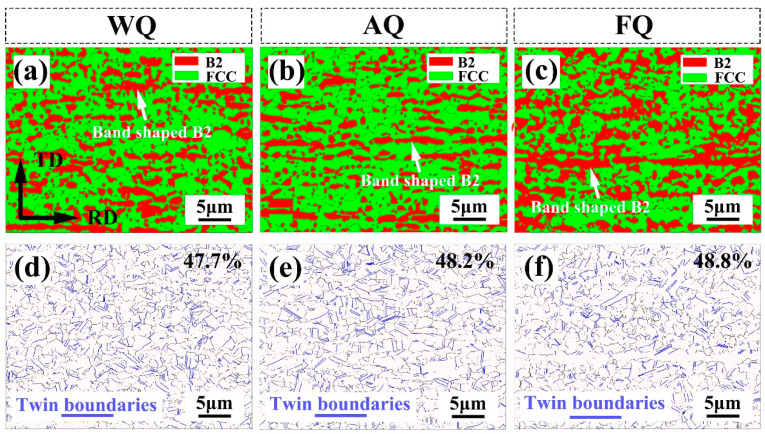
(**a**,**d**) EBSD phase distribution; (**a**,**d**) IPF; (**c**,**f**) grain boundary distribution maps of the (**a**–**c**) WQ sample; (**d**–**f**) AQ sample.

To investigate the effects of the different cooling rates on the microstructure of the Fe-18Mn-10Al-1C-5Ni steel, EBSD analysis was conducted, and the results are presented in Figure 1. Based on the phase distribution map, it is evident that as the cooling rate decreased, the volume fraction of the B2 phase increased. Specifically, the volume fractions of the B2 phases in the WQ (Figure 1a), AQ (Figure 1b), and FQ (Figure 1c) samples were 24.5%, 25.8%, and 41.4%, respectively. Concurrently, the grain sizes of both the austenite and B2 phases also increased with the decreasing cooling rate. In detail, the average grain sizes of the B2 phases in the WQ, AQ, and FQ samples were 1700 nm, 2100 nm, and 3700 nm, respectively, while the average grain sizes of the austenite were 2200 nm, 2700 nm, and 4900 nm, respectively. Additionally, the morphology of the B2 phases underwent changes. Compared to the WQ and AQ samples, the volume fraction of the band-shaped B2 phase in the FQ sample significantly increased, and its size became noticeably coarser. The annealing twins were less influenced by the cooling rate, with their proportion fluctuating between 47.7% and 48.8%, as shown in Figure 1d–f.

**Figure 2:**
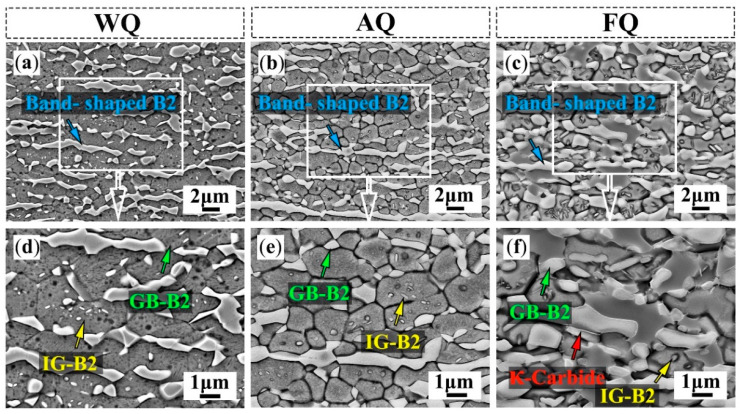
SEM secondary electron images: (**a**,**d**) WQ sample, (**b**,**e**) AQ sample, and (**c**,**f**) FQ sample.

To further investigate the effects of the different cooling rates on the microstructure, the SEM microstructures of the three samples are shown in Figure 2. In these three samples, the polygon B2 phases (GB-B2) and strip-shaped B2 phases (band-shaped B2) are distributed at the grain boundary, as shown in Figure 2a–c. At the same time, small B2 particles can be observed inside the austenite grains (IG-B2) of the WQ sample (Figure 2d) and AQ sample (Figure 2e). However, IG-B2 particles within the grains of the FQ sample are relatively rare (Figure 2f). New precipitates appeared at the grain boundaries of the austenite and B2 phases in the FQ sample. Previous studies [24] on Fe-Mn-Al-C steel have shown that furnace-cooled austenite tends to form κ-carbides at the grain boundaries. According to reports from Choi et al. [14], prolonged aging treatment can lead to the formation of coarsened κ-carbides at the austenite grain boundaries and B2 phase boundaries. Therefore, the precipitated phase appearing at the austenite boundary in the FQ sample may have been κ-carbides.

**Figure 3:**
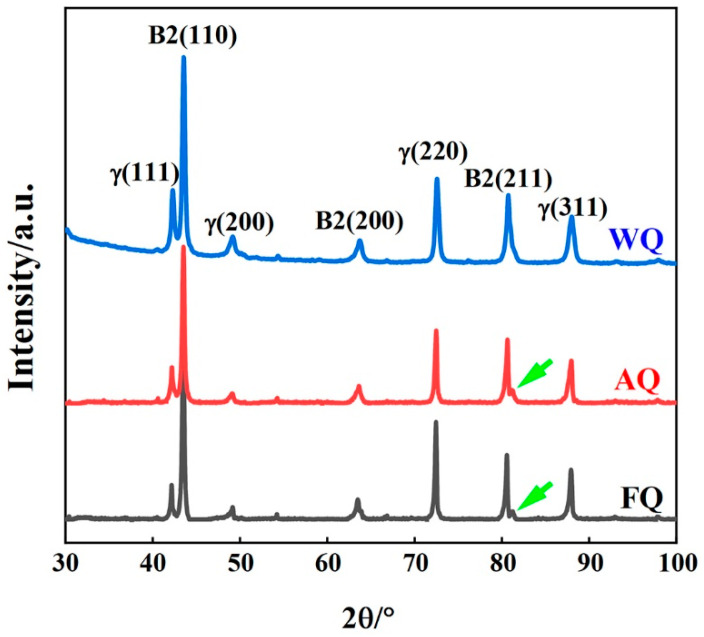
XRD profiles of WQ, AQ, and FQ samples.

To investigate the effects of the different cooling rates on the phase composition of the Fe-18Mn-10Al-1C-5Ni lightweight steel, Figure 3 shows the XRD patterns of the WQ, AQ, and FQ samples. From Figure 3, it can be seen that the XRD patterns of the three samples all exhibit diffraction peaks of austenite (γ) and B2 phases, indicating that the microstructure is composed of austenite and B2 phases. Upon careful analysis, it can be observed that the XRD spectra of the AQ and FQ samples exhibit satellite peaks in the gamma peak, with clear sideband effects, as shown in Figure 3. The results indicate that the Fe-18Mn-10Al-1C-5Ni lightweight steel underwent amplitude modulation decomposition, with the formation of κ-carbide precipitates in the matrix. According to the Bragg diffraction law, as the interplanar spacing (d) increases, the diffraction angle (θ) decreases. Due to the lattice constant of this carbide being greater than that of γ-Fe, the interplanar spacing increased, resulting in a leftward shift of the diffraction peak. Through comparison, it was found that the edge band peak of the FQ sample was the most obvious, and the peak shifted to the left at the largest angle, indicating that the ordered kappa carbide content was the highest at this time. Ordered κ-carbides in the austenite matrix of high-manganese and high-aluminum lightweight steel are formed through amplitude modulation decomposition, exhibiting an L12 structure. [8,11,24,25,26], which can be analyzed by XRD. 

**Figure 4:**
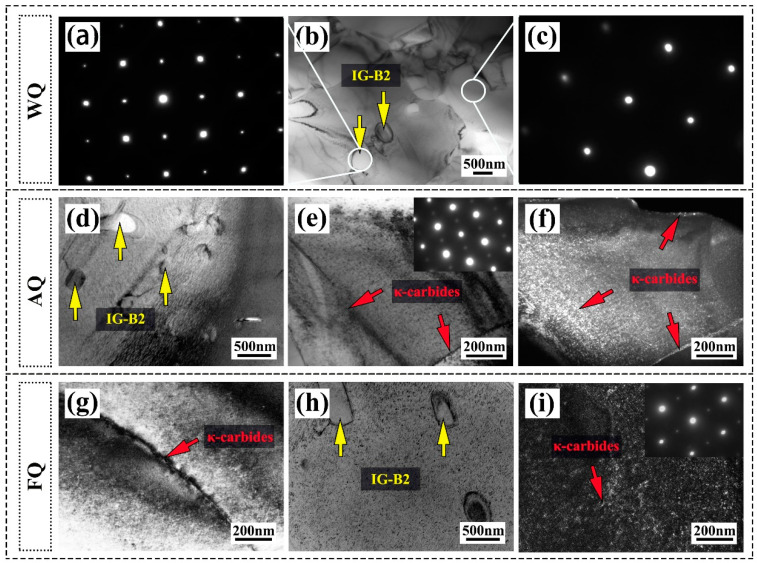
TEM bright-filed and dark-filed micrographs with different precipitates in the (**a**,**b**,**c**) WQ sample; (**d**,**e**,**f**) AQ sample; (**g**,**h**,**i**) FQ sample.

To distinguish the effects of water quenching, air quenching, and furnace quenching on the size and distribution of κ-carbides and B2 phases in this research, we conducted TEM analysis on the three samples, and the results are shown in Figure 4. As shown in Figure 4b, in the WQ sample, B2 particles were formed inside the austenite grains with an average grain size of 230 nm. The diffraction pattern confirmed its ordered BCC crystal structure (Figure 4a). Meanwhile, according to the diffraction pattern from Figure 4c, κ-carbides were not found in the WQ sample. For the AQ samples, there was no significant change in the size of B2 phase (239 nm) within the grains (Figure 4d), but nanoscale κ-carbides appeared at both the grain boundaries and within the grains (Figure 4e,f). As a precipitation hardening phase in Fe-Mn-Al-C lightweight steel, κ-carbides can significantly enhance the tensile strength of the material [11]. In contrast, the B2 phases inside the grains in the FQ sample slightly coarsened and increased in size to 332 nm (Figure 4h). In addition, the κ-carbides inside the grains (Figure 4i) and at the grain boundaries (Figure 4g) were significantly coarsened, especially at the grain boundaries, where the size reached 170 nm. The results indicate that adjusting the cooling method can effectively regulate the size and distribution of B2 phase and κ-carbides precipitates in Fe-18Mn-10Al-1C-5Ni lightweight steel, thereby achieving the optimization of the tensile properties.

### 3.2. Mechanical Properties

**Figure 5:**
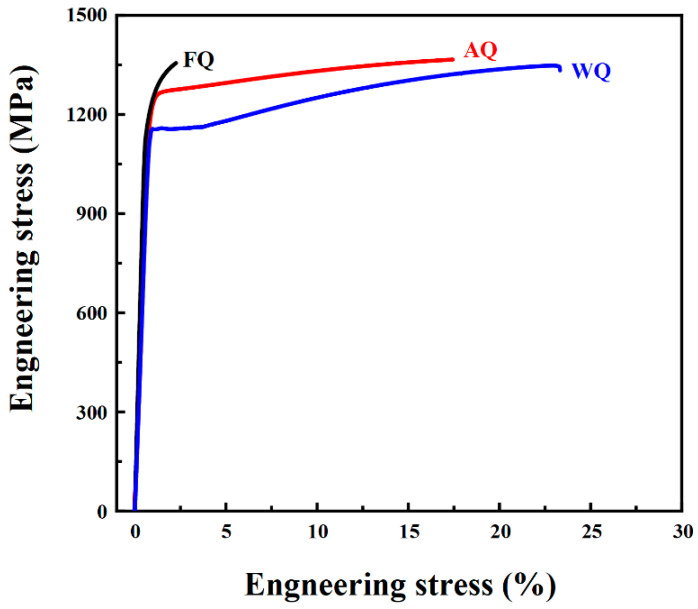
Engineering stress–strain curves of WQ, AQ, and FQ samples.

The engineering stress–strain curves of the WQ, AQ, and FQ samples are shown in Figure 5. Both the WQ and AQ samples exhibit a good combination of ultra-high strength and good ductility, while the FQ sample shows severely deteriorated elongation. Specifically, the yield strength and ultimate tensile strength of the WQ sample were 1094 MPa and 1315 MPa, respectively, with an elongation of 23.3%. The yield strength and tensile strength of the AQ samples further increased, reaching 1232 MPa and 1347 MPa, respectively, while the elongation slightly decreased to 17.4%. It is worth noting that compared to the AQ sample, the yield strength and tensile strength of the FQ sample did not change much at the levels of 1251 MPa and 1357 MPa, respectively, while the elongation rate significantly decreased to 2.3%.

## 4. Discussion

### 4.1. Microstructural Factors Influencing the Tensile Properties

Compared to the WQ sample, after the air quenching treatment, the elongation did not decrease significantly (5.9%), while the yield strength increased from 1094 MPa to 1232 MPa (a 12.6% increment). Hwang et al. [23] have reported that in Fe-21Mn-10Al-1C-5Ni steel, the synergistic effect of the κ-carbides and B2 precipitates on the dislocation pinning significantly enhances the tensile strength. Therefore, it can be inferred that the good balance between strength and elongation achieved by the AQ sample was mainly due to the combined effect of the intragranular IG-B2 particles, intragranular κ-carbides, and intergranular GB-B2 particles. According to the conclusions reported by Kim et al. [17], during tensile deformation, dislocations will be pinned or bowed out at the B2 and austenite interfaces, which greatly enhances the material’s strength. The greater the number of precipitated phases and the finer their size, the greater their contribution to the improvement in the material’s strength [21,27,28]. In this research, the volume fraction of the B2 phase in the AQ sample was as high as 25.8%, and its size was significantly refined, with the average particle sizes of the IG-B2 and GB-B2 being 239 nm and 2100 nm, respectively. Therefore, these high-volume-fraction and refined B2 phases greatly enhance the yield strength through the precipitation hardening mechanism. According to the Hall–Petch equation [23], the smaller the grain size, the higher the tensile strength of the material. The refined austenite grain structure in the AQ sample (with an average size of 2700 nm) provided a significant fine-grain strengthening effect. In addition, as a precipitation hardening phase, κ-carbides can hinder dislocation movement during deformation [25,29,30], thereby significantly improving the tensile strength. The nanoscale intragranular κ-carbides in the WQ sample not only enhanced the material’s strength but also did not cause damage to the elongation [21,25,31]. However, since κ-carbides also appeared at the grain boundaries of the WQ sample, this led to stress concentration and caused some loss of elongation. Nevertheless, because the size of these intergranular κ-carbides did not increase significantly, the elongation of the AQ sample was only reduced by 5.9% compared to that of the WQ sample.

Compared with the WQ and AQ samples, the yield strength and tensile strength of the FQ sample did not increase significantly, but the elongation decreased dramatically to only 2.3%. The severe deterioration in the elongation of the FQ sample mainly stemmed from the coarsened grain boundary κ-carbides (170 nm). The coarse κ-carbides were irregularly distributed at the grain boundaries, which significantly increased the stress concentration at the grain boundaries. Under external forces, it is difficult for these hard and brittle carbides to undergo plastic deformation through dislocation movement or other means to coordinate deformation, resulting in highly localized stress concentration [32,33]. This provides favorable conditions for the initiation and propagation of cracks, ultimately causing the material to fracture under relatively small external forces and significantly reducing the ductility.

### 4.2. Fracture Behavior Investigation and Analysis of the Samples

**Figure 6:**
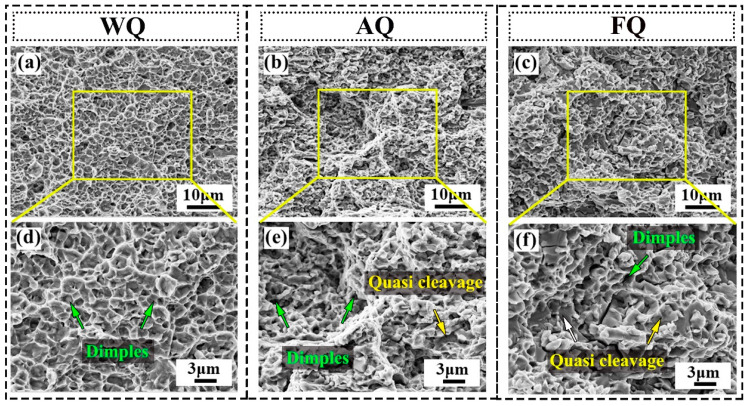
The fractography characterization of tensile samples: (**a**,**d**) WQ sample; (**b,e**) AQ sample; (**c**,**f**) FQ sample.

As is well known, pores and microcracks often nucleate at the interfaces. In this study, compared with the WQ sample, the grain boundaries and phase boundaries of the AQ and FQ samples precipitated κ-carbides, and the newly formed interface may have had an impact on the fracture mode and mechanism. In view of this, SEM analysis was used to characterize the fracture morphology and microstructure of the experimental samples after tensile fracture, as shown in Figure 6. The fracture surfaces of the WQ sample (Figure 6a,d) and AQ sample (Figure 6b) exhibit typical ductile fracture characteristics, consisting of many uniformly sized ductile dimples. In addition to ductile dimples, several regions representing quasi-cleavage fractures can also be found in the fractured AQ specimens (Figure 6e), which may be related to brittle fractures caused by boundary κ-carbides. Compared to the WQ sample, the elongation of the AQ sample only decreased by 5.9%. Therefore, quasi-cleavage fracture within a small range did not lead to a significant decrease in the total elongation of the AQ specimens, which may be attributed to the contribution of austenite to their plasticity. As the cooling rate decreased, the proportion of quasi-cleavage fracture structures in the fracture of the FQ specimens increased significantly (Figure 6c,f). The significant increase in the B2 phase content and the coarsening of κ-carbides are believed to be the reasons for this fracture.

**Figure 7:**
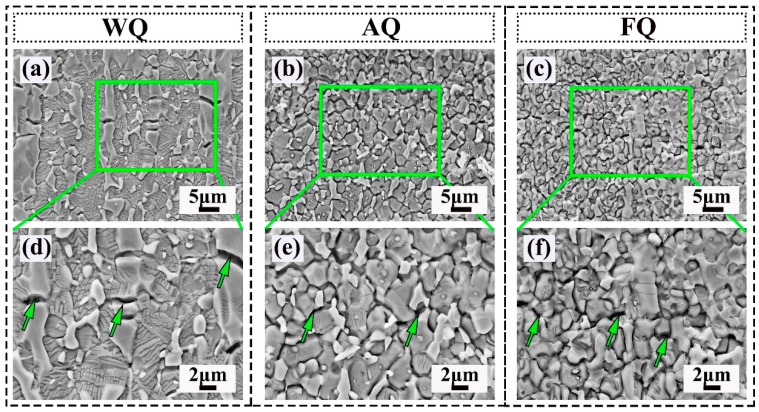
Observations of cavities and microcracks in the tensile samples: (**a**,**d**) WQ sample; (**b**,**e**) AQ sample; (**c**,**f**) FQ sample.

To further investigate the fracture behavior of the Fe-18Mn-10Al-1C-5Ni lightweight steel with B2 phases and κ-carbides, SEM analysis was used to characterize the voids and microcracks. The image near the fracture surface of the fractured sample is shown in Figure 7. It can be concluded that the austenite grain boundaries and B2 interfaces were the main nucleation sites for voids and microcracks (Figure 7a–c), similar to other dual-phase Fe-Mn-Al-C steels [34]. The nucleation of grain boundary voids is related to the deformation ability of the matrix after large deformation [34]. The voids and microcracks at the phase boundary are caused by a plastic mismatch between B2 and austenite. In the AQ samples, κ-carbides precipitated at the austenite grain boundaries and B2 phase boundaries, which may inevitably lead to an increase in the phase boundaries. Therefore, in the case of a similar total elongation, more voids can be observed in the AQ sample (Figure 7b) than in the WQ sample (Figure 7e). In addition, the self-fracture phenomenon of κ-carbides was also discovered, and the epitaxial growth mechanism of kappa carbides resulted in a poor synergistic deformation ability. Due to the weakening effect on the cohesive force of the grain boundaries [35], microcracks can form near the slender strip-shaped κ-carbides at the GB-B2 boundary. In the FQ sample, κ-carbides with a size of 170 nm were formed at the boundary between the B2 and austenite phases, as shown in Figure 7f. In addition, due to favorable growth conditions, the size and width of the κ-carbides significantly coarsened [36]. According to the weakest-link theory model, the fracture probability of κ-carbides will increase with their quantity and size. When κ-carbides are continuously connected, the probability of fracture further increases, as they can be considered long κ-carbides. Therefore, they were the cause of the many voids and microcracks in the FQ sample. These microcracks will rapidly propagate along the grain boundaries or phase boundaries throughout the entire specimen, ultimately leading to fracture at very small strains.

## 5. Conclusions

This study focused on Fe-18Mn-10Al-1C-5Ni lightweight steel and deeply explored the influence mechanisms of different cooling methods (i.e., water quenching, air quenching, and furnace quenching) on the precipitation behavior of the B2 phases and κ-carbides. Additionally, the organization evolution processes of these precipitation phases were analyzed in detail, and the intrinsic relationship between them and the mechanical properties and fracture behavior was revealed. The Fe-18Mn-10Al-1C-5Ni lightweight steel achieved excellent tensile properties through the air quenching method, demonstrating great potential for application in the field of automotive lightweighting. The main research conclusions are summarized as follows:

(1) The microstructure of the WQ sample comprised austenite, grain boundary B2 (GB-B2), and intragranular B2 (IG-B2). The B2 phase had a volume fraction of 25.8%, with the average grain sizes of the GB-B2 and IG-B2 being 1700 nm and 230 nm, respectively. The yield strength and tensile strength of the WQ sample were 1094 MPa and 1315 MPa, respectively, accompanied by an elongation of 23.3%;
(2) Compared with the WQ sample, the IG-B2 particles in the AQ sample did not change significantly (239 nm), while nano-sized κ-carbides appeared at the grain boundaries and within the grains. After the air cooling treatment, the yield strength and ultimate tensile strength of the AQ sample were significantly improved to 1232 MPa and 1347 MPa, respectively, while ensuring that the ductility was not seriously deteriorated (17.4%). This excellent strength–plasticity balance can be mainly attributed to the synergistic effect of GB-B2, IG-B2 particles, and nano-sized κ-carbides in austenite;
(3) Compared with the WQ and AQ samples, the IG-B2 particles in the FQ sample became coarser (332 nm). Meanwhile, the intragranular κ-carbides also coarsened, especially at the B2–austenite interfaces, where new κ-carbides grew to 170 nm. Furnace quenching led to a severe deterioration in the elongation during the tensile deformation process, and the κ-carbides at the phase boundaries promoted the nucleation of voids and microcracks.

## Data Availability

The original contributions presented in this study are included in the article. Further inquiries can be directed to the corresponding author.

## Abbreviations

The following abbreviations are used in this manuscript:

WQ	Water quenching
AQ	Air cooling
FQ	Furnace cooling
